# Crown Preservation of the Mandibular First Molar Tooth Impacts the Strength and Stiffness of Three Non-Invasive Jaw Fracture Repair Constructs in Dogs

**DOI:** 10.3389/fvets.2015.00018

**Published:** 2015-07-17

**Authors:** Charles Lothamer, Christopher John Snyder, Sarah Duenwald-Kuehl, John Kloke, Ronald P. McCabe, Ray Vanderby

**Affiliations:** ^1^Department of Surgical Sciences, School of Veterinary Medicine, University of Wisconsin-Madison, Madison, WI, USA; ^2^Department of Orthopedics and Rehabilitation, University of Wisconsin-Madison, Madison, WI, USA; ^3^Department of Biostatistics and Medical Informatics, School of Medicine and Public Health, University of Wisconsin-Madison, Madison, WI, USA

**Keywords:** non-invasive, fracture, repair, mandible, dogs, composite, strength, stiffness

## Abstract

Repairing mandibular body fractures presents unique challenges not encountered when repairing long bones. Large tooth roots and the presence of the inferior alveolar neurovascular bundle limit safe placement for many types of orthopedic implants. Use of non-invasive fracture repair methods have increasingly become popular and have proven safe and effective at achieving bone healing. Non-invasive fixation constructs have not been tested in dogs using cantilevered bending. Furthermore, non-invasive fracture repair constructs have not been tested at the location of a common fracture location – the mandibular first molar tooth (M1). The objectives of this study were to test the strength and stiffness of three non-invasive mandibular fracture repair constructs and to characterize the impact that tooth crown preservation has on fixation strength for fractures occurring at the M1 location. Specimens were assigned to three treatment groups: (1) composite only, (2) interdental wiring and composite (IWC), and (3) transmucosal fixation screw and composite. For each pair of mandibles, one mandible received crown amputation at the alveolar margin to simulate the effect of crown loss on fixation strength and stiffness. Regardless of the status of crown presence, IWC demonstrated the greatest bending stiffness and load to failure. With the crown removed, IWC was significantly stronger compared to other treatments. All fixation constructs were stiffer when the tooth crown was preserved. In fractures at this location, retaining the tooth crown of M1 significantly increases stiffness of interdental wiring with composite and transmucosal screw with composite constructs. If the crown of M1 was removed, IWC was significantly stronger than the other two forms of fixation.

## Introduction

Mandibular fractures are the most commonly occurring maxillofacial fractures in veterinary patients (1–3). Lopes et al. reported that 90% maxillofacial injuries in their canine study population were mandibular fractures (1). Kitshoff et al. and Lopes et al. reported mandibular fractures tended to occur in the molar region of 41.5–47.1% (1, 2) of cases. In addition to restoring mechanical function through rigid support and fixation, fracture repair should reduce pain and optimize bone healing. Fractures involving the mandible also require attention to the restoration of occlusion and minimization of injury to neurovascular structures (4), which can complicate repair.

Non-invasive fracture repair techniques do not require surgical exposure of the fracture site and minimize risk of damaging or disrupting anatomic structures such as tooth roots or vessels. Non-invasive techniques have gained popularity due to extensive experience with clinical application of dental composites in veterinary medicine (5–11). The use of human dental composites to create intraoral splints in dogs and cats has proven safe (5), strong (4), and clinically effective (6–11) when used as an alternative to open reduction and internal fixation. Through application of the tension band principle, placement of a fixation device along the oral surface of the mandible generates a reciprocal compressive force along the ventral cortex (12). Forces may concentrate in the area of the first molar tooth (M1) due to decreased mandibular bone height over the distal root and buccal cortical bone thinning over the mesial root (13). Due to the relative frequency of fractures occurring at this location and challenges associated with repair in this area, maximizing the mechanical effectiveness of fixation devices is an important consideration. Furthermore, non-invasive (intraoral) repair methods at this location are limited by the number of available teeth distal to the fracture and these teeth have relatively small crown surface areas for fixation attachment.

Teeth suffering periodontitis or endodontic disease prior to fracture have previously been recommended to be extracted (12). Frequent involvement of M1 in mandibular fractures forces a clinical decision to be made as to whether or not the tooth should be extracted or receive advanced endodontic treatment at the time of fracture repair (14–16). If a fracture line involves the tooth root, maintaining that structure presumably contributes to stabilization of the fracture and improves healing (17). By maintaining the tooth in the fracture line, stability of the fracture is improved and the correct alignment and occlusion are maintained (14, 15, 18); however, the impact of maintaining the tooth crown on fixation device strength and stiffness is unknown and has not been reported.

The purposes of this study were twofold: (1) to compare the strength and stiffness between three non-invasive mandibular fracture fixation constructs using cantilevered bending and (2) to compare the impact on strength and stiffness that retention of the crown of M1 had on the three non-invasive fixation techniques.

## Materials and Methods

The mandibles from 32 deceased Beagle dogs with adult dentition (>8 months of age) were obtained for this study. All mandibles were grossly examined for complete dentition and normal tooth morphology extending from the canine through the third molar teeth. Periodontal probing was performed and intraoral dental radiographs^1^ were obtained for the aforementioned teeth. Specimens were excluded from the study if dentition was incomplete and if the periodontal disease score was >stage 1 (gingivitis only) (19). Specimens were excluded if there were evidence of root fracture, internal or external tooth resorption, horizontal or vertical bone loss, periapical pathology, or open apices indicating immature animals or tooth non-vitality. Mandibles were disarticulated and all soft tissues were removed with the exception of free and attached gingiva. Paired mandibles were separated at the symphysis and wrapped in a distilled water-soaked paper towel and stored in individually labeled plastic bags.

Paired specimens were randomly assigned to one of three treatment groups: composite only (CO), transmucosal screw/composite (TSC), or interdental wiring/composite (IWC). Within each treatment group, specimens were randomly assigned to a group designated for removal of the crown of M1 to simulate the effect that tooth crown removal may have on mechanical properties of fixation. Eight paired specimens were designated as controls (CNTL) and were randomly assigned to have one mandible of each pair to undergo removal of M1.

Specimens were frozen (−20°C) until fixation and biomechanical testing. All mandibles underwent a maximum of one freeze–thaw cycle. No specimens were frozen following dental scaling and polishing. Whenever thawed, specimens were wrapped with wet paper towels to maintain hydration.

**Figure 1:**
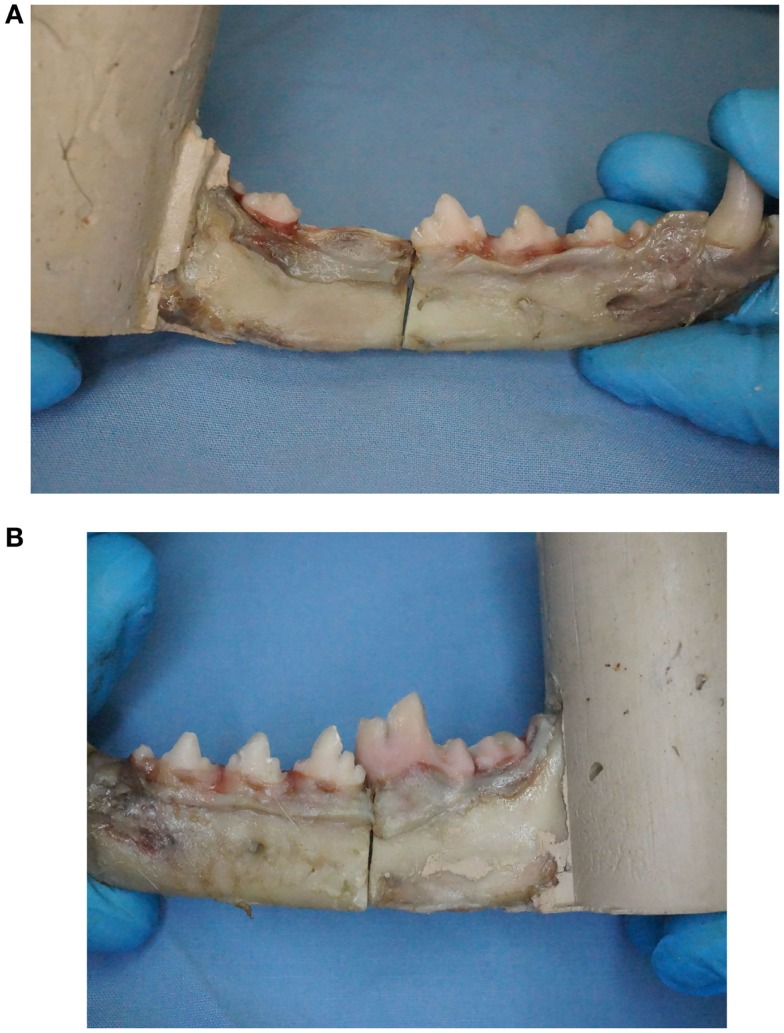
**(A)** One mandible per pair was randomly assigned to undergo removal of mandibular first molar tooth crown to mimic tooth crown loss similar to what occurs with tooth extraction. **(B)** Maintaining the first molar tooth crown provides an additional anchorage point for fixation proximal to the fracture when using non-invasive fracture repair techniques.

An osteotomy was created mesial to M1 and perpendicular to the long axis of the mandibular body on all specimens except CNTL. A surgical oscillating saw^2^ with a thin kerf blade^3^ was used to create the osteotomy using a custom jig for positioning. For the specimens designated the crown removal group, crown amputation was performed to the level of the alveolar margin using a #700 crosscut fissure bur^4^ on a water-cooled, high-speed hand piece (Figure 1). Tooth crowns for all treatments (CO, TSC, IWC) were ultrasonically scaled and polished with pumice. Rinsing of specimens was performed with distilled water.

**Figure 2:**
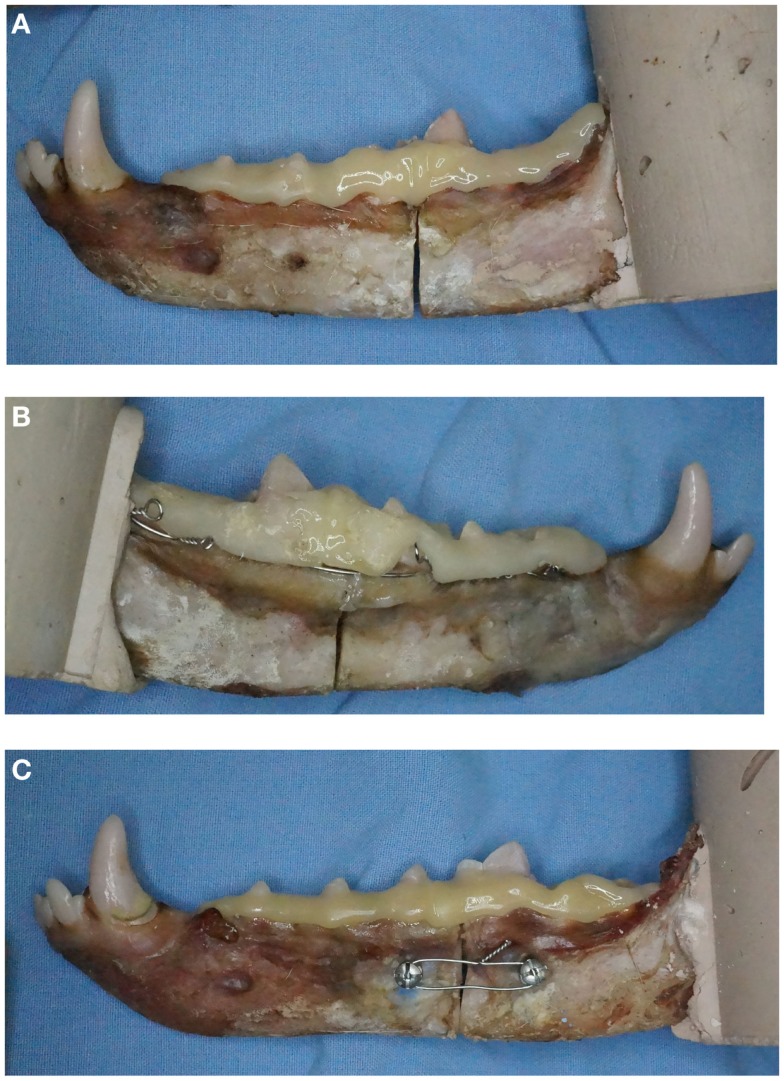
**Examples of the three fracture repair techniques applied to specimens with intact first molar crowns**. Specimens demonstrating **(A)** composite only (CO), **(B)** interdental wiring and composite (IWC), and **(C)** transmucosal screw and composite (TSC) are shown.

At the time of fixation application, mesial and distal segments were stabilized using a 2″ C-clamp placed over the osteotomy site. The interdental wiring treatment (IWC) received a Stout loop-wiring pattern (5) using 24-gage stainless steel orthopedic wire^5^. Consistent with a previously described mechanical model for strength testing, interdental wiring was performed from the third molar tooth through the first premolar tooth (4). In the TSC treatment, pilot holes were drilled bicortically and perpendicular to the buccal cortical bone surface using an electric drill and 1.5 mm drill bit. Holes were drilled 1 cm mesial and distal to the osteotomy and 1 cm ventral from the alveolar margin. If screw placement was anticipated to contact root structure, pilot hole placement was modified 2 mm mesial or distal. Two-point-zero millimeter self-tapping IMF screws^6^^,^^7^ were secured bicortically using a hand-operated screwdriver. Twenty-four-gage orthopedic wire^5^ was secured between screws using ligature holes in the screw head. Compression was achieved by twisting the wire ends until the wire was taut or until there was evidence of gapping of the lingual cortical plate (Figures 2A–C).

For all treatments, the crowns of all teeth extending from the first premolar through third molar were etched for 20 s with 38% phosphoric acid^8^. Crown surfaces were rinsed and lightly air-dried with compressed nitrogen. Self-curing bis-acryl composite^9^ was applied to crown surfaces from the first premolar tooth through the third molar tooth. Composite was expressed from an application gun using a two-part cartridge with automixing tip and applied in a fashion that was clinically appropriate for the size of the specimen by a single investigator (CL). Stable fixation was confirmed with assessment of the apparatus following removal of the C-clamp.

Specimen weight, time of application of fixation steps, and fixation weight were recorded. For consistency, a single investigator (CL) performed IWC application. The ramus of each specimen was embedded in a self-curing methacrylate acrylic^10^ using a custom mold. At time of embedding, the body of each mandible was placed on a 10° incline ramp relative to the mold to account for flex during biomechanical testing. Following curing, the specimens were maintained in a distilled water-soaked paper towel, placed in a plastic bag, and stored at room temperature until mechanical testing.

### Mechanical testing

**Figure 3:**
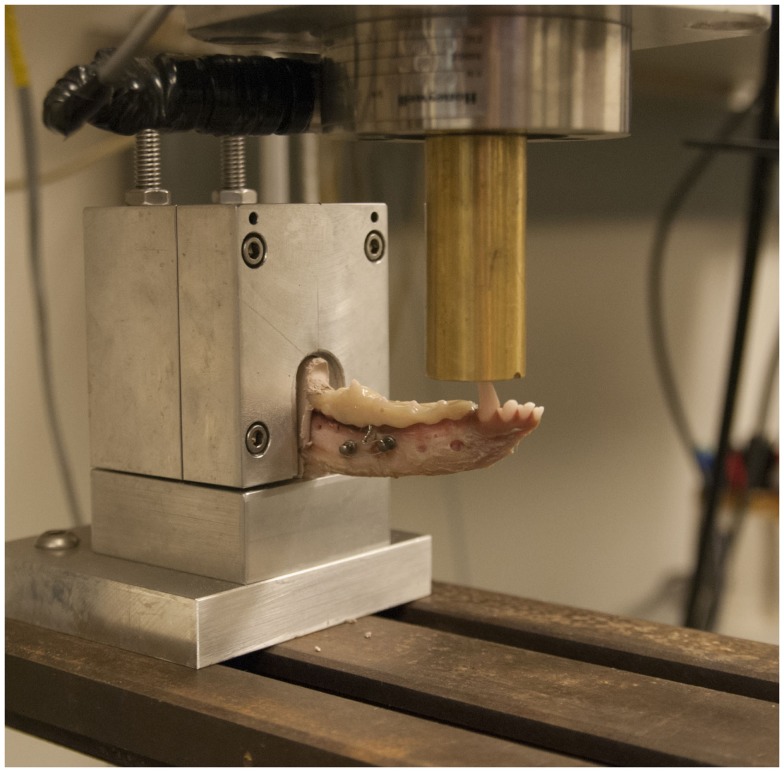
**Specimen testing setup demonstrating the specimen placed in a custom grip and point force cantilevered bending being applied to the canine tooth with a servohydraulic testing system**.

Testing of the structural stiffness and load–displacement behavior was measured using a servohydraulic testing system^11^ and a custom jig designed to support the mandible during cantilevered bending (Figure 3).

Specimens were loaded into the testing jig and force was applied to the canine tooth cusp tip. A 1000 lb load cell was used to record the applied force. Force was applied at a speed of 10 mm/min. The distance from the jig to the point of force application was recorded in order to calculate the moment arm.

### Data analysis

**Figure 4:**
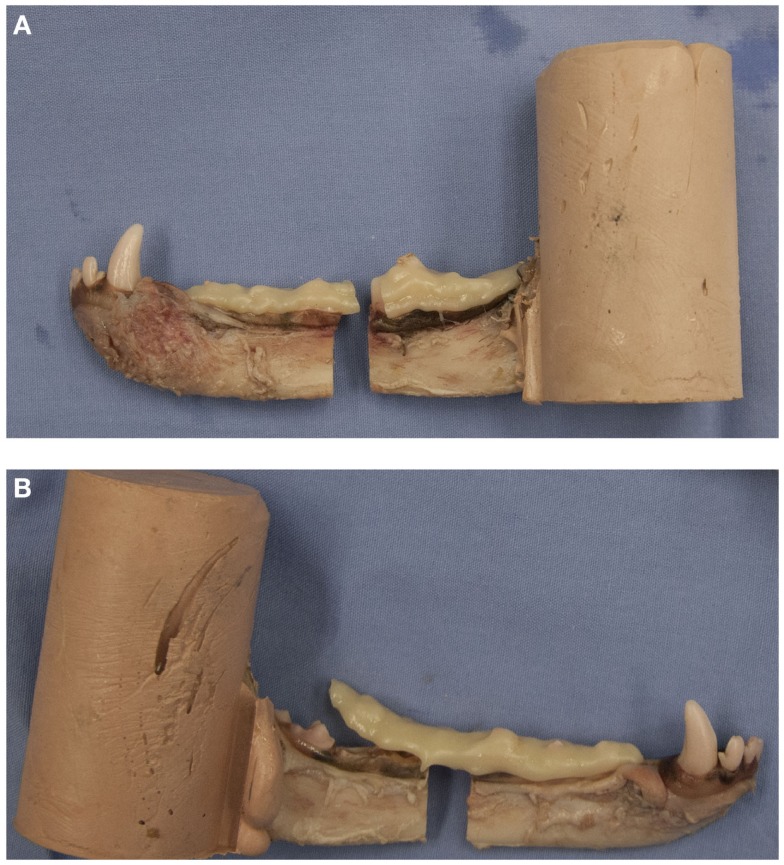
**Examples of a pair of potted hemi-mandibles receiving composite-only treatments**. The left mandible **(A)** received composite fixation and demonstrates cohesive failure of the fixation device. The right mandible **(B)** received crown removal and demonstrates adhesive failure of the fixation device.

Failure was defined as the point at which cohesive failure (construct breakage) or adhesive failure (construct separation from tooth structure) occurred (Figures 4A,B). For CNTL, the point of failure was defined when fracture of the bony tissue occurred.

Graphs were plotted of the acquired data from the load cell and the ram displacement (millimeter), displaying load (Newton) vs. displacement (millimeter). Ultimate strength was defined as the point at which the structure was under maximal load. Stiffness was calculated by determining the slope of the linear segment of the load–displacement curve (Newton per millimeter). The type of failure – cohesive, adhesive, or tissue- was noted for all specimens.

### Statistical analysis

Data were analyzed to compare the ultimate strength and stiffness for each fixation treatment and between crown removal groups. An evaluation was performed between fixation techniques to evaluate the effect of the presence of the crown of M1. For each fixation treatment, the presence or absence of the tooth crown was analyzed to determine whether a difference in strength or stiffness existed. The following tests and comparisons were performed (with *p* ≤ 0.05 for significance). Robust rank-based linear mixed models were fit using a sandwich estimator to estimate SE (20). Specimen was included as a random effect while crown removal and technique were included as fixed effects. Contrasts for specific effect sizes were estimated as well as Wald-type confidence intervals or tested based on Wald-type tests. A model for the control specimens was fit separately from the fracture specimens. Interaction plots based on the Hodges–Lehmann estimate (HLE) of location were used to assess treatment interaction (21). In the presence of interaction, effects within a level were estimated. When no interaction was apparent, inference was done on main effects. Analyses were run using R^12^.

## Results

Time for application of wire, screw, and composite during fixation steps, and weight of fixation weight were recorded (Table S1 in Supplementary Material). Time of composite application was consistent between treatments. Time of IWC application did not vary regardless of the tooth crown group; however, overall time taken to apply fixation of the IWC treatment was greater than the time required for TSC treatment (2.5× greater for crown removed and 2.8× greater for crown present groups). Specimen age and weight were recorded (Table S2 in Supplementary Material) and did not vary between treatments.

**Figure 5:**
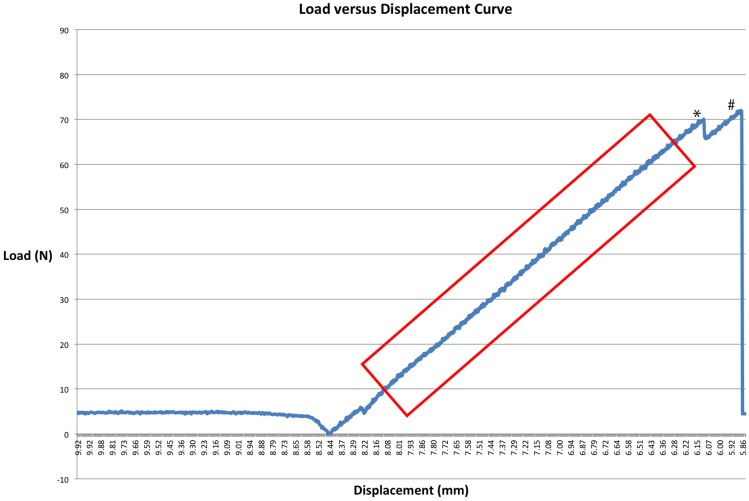
**A load–displacement curve of a CO specimen**. * denotes a point where load briefly decreased before ultimate failure occurred. This may suggest clinical failure of the fixation construct, a point at which point the construct may demonstrate instability. # denotes ultimate failure. The box demonstrates a linear segment of the graph used to calculate the slope to determine stiffness.

Ultimate failure load was determined by bone fracture for CNTL mandibles. One control mandible did not register an ultimate failure load point due to the specimen exceeding the displacement and load limits of the test setup. Ultimate failure load for all treatments was defined as the point of greatest load before failure. Several treated specimens’ results registered a slight decrease in load before experiencing ultimate failure. All specimens contained a significant linear segment enabling stiffness calculation as the slope of the best-fit line (Figure 5). Cohesive failure (fracture of the construct) occurred in 72.9% (35/48) of treatment specimens. The remaining 27.1% (13/48) specimens failed by adhesive failure (decrease in load resulting from separation of the apparatus from tooth structure).

**Figure 6:**
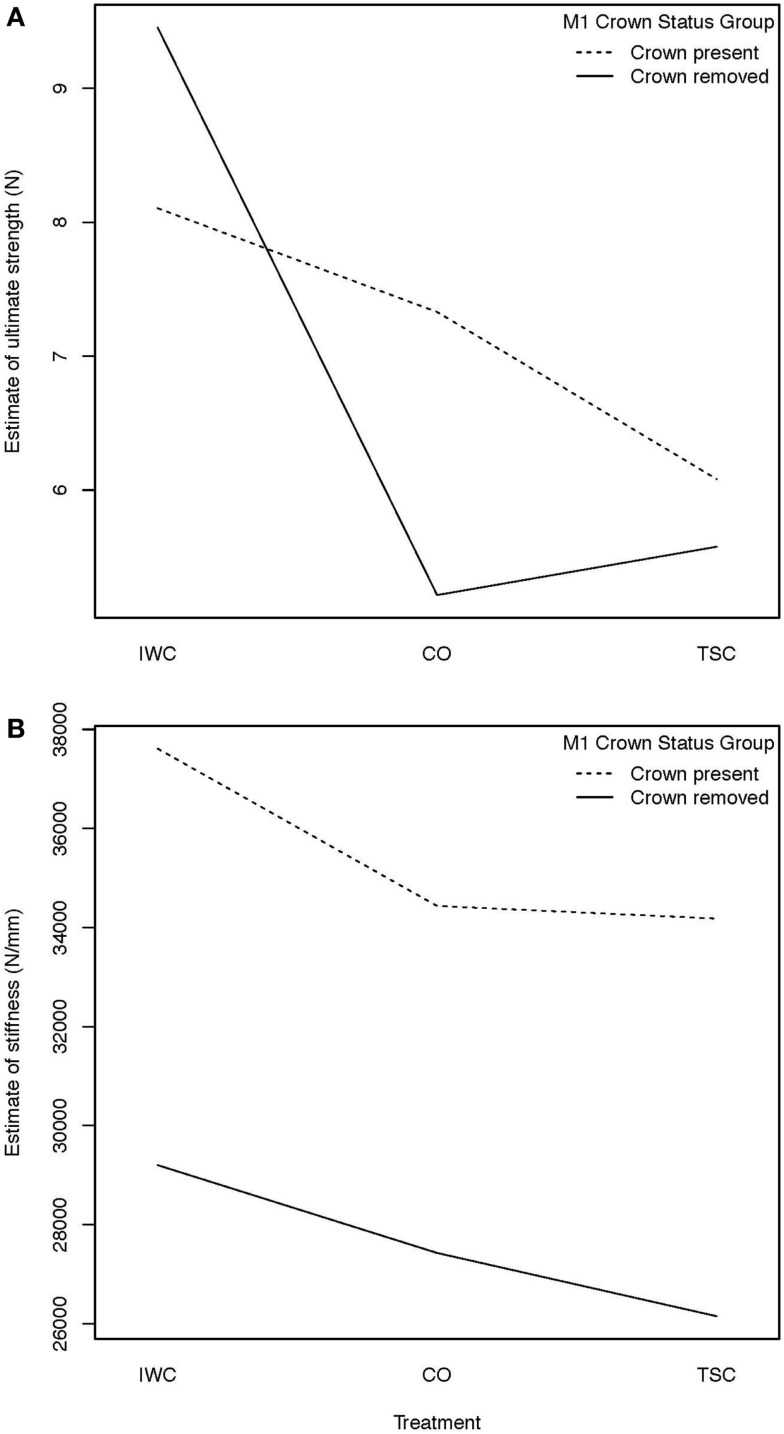
**(A)** Hodges–Lehmann estimate to assess treatment interaction, treatment interaction with crown status effect on strength: an interaction from the effect of removal of the crown of M1 on ultimate strength is demonstrated by the intersection of the lines in the graph. Removal of the crown of M1 was found to result in increased mean ultimate strength of the IWC treatment and a mean decreased ultimate strength of the CO and TSC treatments. **(B)** Hodges–Lehmann estimate to assess treatment interaction, treatment interaction with crown status effect on stiffness: when evaluating the effect of removal of the crown of M1 on stiffness, no interaction is apparent from the absence of intersection between plots. Further statistical evaluation is necessary to demonstrate the mild (inapparent) interaction. This effect is noted when evaluating the strong effect crown status has on stiffness of the IWC and TSC constructs.

The impact of the presence or absence of M1 on ultimate strength and stiffness were evaluated for each treatment. Comparison within treatments was demonstrated using a HLE to demonstrate treatment interaction (Figure 6). An interaction in the graph of ultimate strength is apparent as demonstrated by the intersection of lines relative to crown status as compared by treatment. An interaction in the graph of stiffness is not apparent when comparing estimate of main effects.

To estimate the effect of the loss of crown structure, contrasts based on a mixed model analysis were used (Table 1). Results indicate that when testing the effect of M1 crown removal, the ultimate strength within each treatment was not significantly different (IWC *p* = 0.38, CO *p* = 0.16, TSC *p* = 0.73). Treatment comparisons of means within crown groups provide direct measure of the relative impact crown presence has on different pairs of fixation. When the crown of M1 is present, the ultimate strength compared between fixation techniques was not different (IWC vs. CO = 0.68, IWC vs. TSC = 0.29, CO vs. TSC = 0.59). In the crown removed groups, IWC was shown to be significantly stronger than both CO (*p* = 0.014) and TSC (*p* = 0.034) treatments. Although not statistically significantly different, the mean ultimate strength of the CO treatment and that of the TSC treatment were greater when the crown was intact (CO: crown present = 7.5 Nm, crown removed = 5.3 Nm, TSC: crown present = 6.4 Nm, crown removed = 5.8 Nm).

**Table 1:** **Ultimate strength of various forms of fixation and impact of crown removal are demonstrated**.

Ultimate strength of fixation techniques compared by crown status group comparisons within treatments								
Treatment	Mean (Nm)	Crown present group	95% CI	Mean (Nm)	Crown removed group	95% CI	Comparison of means within treatments	

		SE			SE		*p*-value	
IWC	8.4	1.5	5.3–11.5	9.6	1.3	6.9–12.2	0.38	
CO	7.5	1.5	4.3–10.6	5.3	1.2	2.8–7.8	0.16	
TSC	6.4	1.3	3.6–9.1	5.8	1.3	3.2–8.5	0.73	
Control	30.8	5.6	17.9–43.7	32.6	5.6	19.7–45.6	0.43	

**Estimation for effect of crown status on ultimate strength between treatments**								

	**Estimation (Nm)**	**Crown present group**	**95% CI**	***p*-value**	**Estimation (Nm)**	**Crown removed group**	**95% CI**	***p*-value**

		**SE**				**SE**		

IWC vs. CO	0.9	2.2	−3.7 to 5.5	0.68	4.3	1.6	0.9–7.6^a^	0.014
IWC vs. TSC	2	1.8	−1.8 to 5.8	0.29	3.7	1.7	0.3–7.1^a^	0.034
CO vs. TSC	1.1	2.0	−3.0 to 5.1	0.59	−0.6	1.3	−3.3 to 2.2	0.68

*Within treatments, no difference existed between crown status groups. Treatment comparison of means within crown groups evaluates the difference in mean ultimate strength between two treatments. These results demonstrate that if the crown is removed, a significant difference exists between the ultimate strength of the IWC and CO treatments indicating IWC would be the stronger technique. Likewise, if the crown was removed and treatment choices were IWC or TSC, IWC would be significantly stronger than TSC. If the crown were removed there would be no difference in strength between CO and TSC. If the crown were present, there was no difference in strength between any comparisons of treatments*.

*^a^Denotes significant increase in difference between groups based on a Wald test*.

When comparing stiffness using an estimation of means (HLE), an overall trend existed demonstrating that the crown removal groups were less stiff for each treatment (Figure 6). Stiffness of fixation treatments compared by crown status group within treatments demonstrated a significant reduction in stiffness in both the IWC (*p* = 0.03) and TSC (*p* = 0.002) treatments (Table 2). Comparisons of the effect of crown status on stiffness between treatments revealed no difference between treatment techniques.

**Table 2:** **Comparison of stiffness (N/mm) between treatments with respect to crown presence**.

Stiffness of fixation techniques compared by group comparisons within treatments								
Treatment	Mean (N/mm)	Crown present group	95% CI	Mean (N/mm)	Crown removed group	95% CI	Comparison of means within treatments	

		SE			SE		*p*-value	
IWC	38156.9	3901.9	30085.3–46228.5	29091.4	4251.0	20297.5–37885.4	0.03^a^	
CO	33300.1	4619.6	23743.7–42856.5	26902.6	3605.9	19443.2–34362.0	0.26	
TSC	33890.3	3513.4	26622.2–41158.4	25539.7	3899.0	17474.0–33605.3	0.002^a^	
Control	44520.5	7537.3	27139.4–61901.7	43506	7537.3	26124.9–60877.1	0.96	

**Estimation for effect of crown status on stiffness between treatments**								

	**Estimation (N/mm)**	**Crown present group**	**95% CI**	***p*-value**	**Estimation (N/mm)**	**Crown removed group**	**95% CI**	***p*-value**

		**SE**				**SE**		

IWC vs. CO	4856.8	5629.3	−6788.4 to 16502.0	0.40	2188.8	4879.0	−7904.2 to 12281.9	0.66
IWC vs. TSC	4266.6	5087.8	−6258.2 to 14791.4	0.41	3551.8	5827.1	−8502.4 to 15606.0	0.55
CO vs. TSC	−590.2	5252.4	−11455.6 to 10275.2	0.91	1362.9	4416.0	−7772.3 to 10498.2	0.76

*Within IWC and TSC treatments, crown removal resulted in a significant decrease in stiffness. Crown status did not result in a change in stiffness for the CO treatment. Estimating for the effect of crown status on stiffness between treatments results in no difference between pairs of treatments based on crown status*.

*^a^Denotes significant difference between groups based on a Wald test*.

The presence of the tooth crown had a significant overall effect (*p* = 0.003) on stiffness across all treatments when comparing an estimate of means (Table 3).

**Table 3:** **Crown presence had a strong positive effect (increase) in overall stiffness for all treatments when considered in aggregate**.

The overall effect of crown removal on stiffness for all treatments				
	Estimation	SE	95% CI	*p*-value
Effect of crown removal	−7937.9	2362.9	−12825.9 to −3049.8	0.0027

No significant difference existed in ultimate strength or stiffness in the control groups regardless of tooth crown presence.

## Discussion

Many variables contribute to clinical decision making in mandibular fracture repair. The purpose of this study was two-part; to evaluate the strength and stiffness of three non-invasive fracture repair techniques using cantilever bending, and to evaluate the impact that crown removal may have on strength and stiffness of those constructs.

Results of this study show maintaining the crown of M1 results in overall increased stiffness across treatments; however within each treatment group, the increase in stiffness was not always significant. An increase in stiffness was significant for the IWC and TSC forms of fixation when the crown was present. The increased stiffness when the crown is present is likely associated with the tooth crown’s involvement with composite’s contribution to stiffness of the construct. Short distances (crown-to-crown) spanned by composite results in increased construct stiffness and resistance to bending. Removal of the tooth crown resulted in the greatest reduction in the mean stiffness of the IWC construct. The structural stiffness of a material is directly affected by the thickness of that material (22). An attempt was made to use a similar volume of composite when applying the splint to all specimens. Additional composite was necessary to occupy the space of the missing molar tooth crown; however, despite composite being thicker at this location, the overall stiffness of the construct decreased. The stiffness of a material is calculated from the slope of the load–displacement curve (22), and therefore if a material undergoes a greater amount of displacement to reach the same load, the stiffness will be lower. For this study, the rate of displacement was fixed and directly related to time. This single-load testing model suggests that efforts to retain dental crowns for incorporation into the composite repair constructs will result in a stiffer construct.

When testing with four-point bending, acrylic splints incorporating Erich arch bar interdental wiring technique showed an increased ultimate strength and stiffness compared to acrylic splints used with or without other wiring techniques (Stout Loop) (4). In that study, Stout interdental wiring with acrylic demonstrated an insignificant increase in strength and insignificant decrease in stiffness compared to acrylic alone (4). Test results using cantilevered bending concurred with the previous study by demonstrating no significant difference in strength or stiffness between constructs when M1 crown is present. With the M1 crown removed, cantilevered bending demonstrated IWC having significantly increased load to failure compared to other constructs. Cantilevered bending, as a testing methodology, replicates similar biomechanical forces experienced by the mandible during mastication.

Direct bone healing (vs. indirect bone healing) occurs in the presence of anatomic fracture reduction and rigid fixation (23). Maintaining the crown of M1 increases the stiffness significantly for the IWC and TSC constructs which demonstrates a more rigid construct. A less rigid construct risks increased motion which results in greater indirect bone healing and associated callus formation (9) with delayed return to function. Whenever clinically possible, maintaining the crown of M1 when managing fractures at this location should result in a stiffer construct through short distances spanned by composite.

Our results show that when M1 crowns were removed, the IWC construct was significantly stronger compared to CO and TSC constructs. Therefore, in clinical situations where the crown of M1 must be removed, the strongest anticipated construct tested will be IWC. It is unknown how predictable removal of an entire tooth root has on testing since root extraction creates an inherent instability in the reduction of the fracture fragments. Further investigation in this area may help discern how various dental treatment options may impact fixation strength for fractures occurring at this location.

An interaction was evident in both strength and stiffness when comparing estimates within crown status groups. Mean ultimate strengths decreased in the CO and TSC treatment groups when the crown was not present but mean ultimate strength increased in the IWC treatment group. The wire’s contribution to ultimate strength is greatest in the IWC construct when the tooth crown is missing. When comparing the effect of crown status on stiffness, IWC and TSC were significantly stiffer when the crown was present. Further studies are needed to determine exactly how the wire of the IWC and TSC treatments may contribute to the stiffness. Removal of the crown of M1 resulted in increased ultimate strength but decreased stiffness for IWC. Under this condition, the wire’s contribution to construct strength is greater than the absence of the tooth crown decreasing construct stiffness. The specific relationship between the wire and composite, and contribution to ultimate strength is likely dynamic and was not investigated in this study. Regardless of treatment selected, stiffness of each repair should be expected to decrease with removal of the tooth crown. Interaction was likely unapparent in the graph due to the strong difference in stiffness of IWC, and TSC between crown status groups. This is further evidenced by the significant effect of crown status on stiffness when estimated across all treatments (Table 3, *p* = 0.003). Maintaining the tooth crown may be clinically relevant for the optimization of bone healing through a stiffer construct’s ability to minimize micromotion.

Difficulties in applying compressive force at the osteotomy site for the CO treatment may contribute to the reported low values for ultimate strength. The CO treatment was the only treatment where compression was not created by wire at the time of composite placement. Fixation devices are strongest in tension and any fracture gapping may introduce dynamic movement during force application and result in failure. Care must be taken when tightening the interdental wire to prevent fracture gaping at the ventral cortex. When the mandible is placed under load, and there is an absence of gapping, neutralization occurs between tension forces created along the alveolar surface and compression generated at the ventral cortex.

When used to apply maxillomandibular fixation in humans, transmucosal screws are placed along the buccal alveolar margin. When experimentally applied across a fracture in this study, tightening the wires generates a compressive force on the buccoalveolar surfaces which risks gap formation along the ventrolingual surface. Tension forces created by wire on the buccal surface may have introduced a dynamic force distribution during loading and contributed to weakening of the construct. This differs from compressive forces generated along the alveolar surface by the Stout Loop interdental wiring technique, which are countered by tension forces applied during loading. If TSC could be shown to be biomechanically advantageous in predetermined situations over IWC constructs (various fracture configurations or combinations of missing teeth), the use of transmucosal screws could be a time-saving measure and decrease the risk for wire sticks to the patient and the surgeon during application.

Transmucosal screw and composite fixation did not show equivalent ultimate strength to IWC fixation in this study. TSC fixation may still offer a beneficial clinical application in edentulous patients or those with advanced periodontal disease lacking healthy available anchorage teeth. In cases where a significant loss of dental tissue exists with which to secure an interdental wire, the IMF screw could be a useful tool through which stabilization of the fracture can be achieved. *In vivo*, stabilization of the rostral mandible by the contralateral mandible and the pull by musculature in the intermandibular space will increase or decrease buccolingual stabilization depending on obliquity of the fracture. Of the constructs tested here, we believe that TSC fixation would be best at counteracting any forces of distraction that may result in the buccal cortical gapping as a result of the pull by the intermandibular muscles.

A larger sample size may have shown a difference in ultimate strength for the CO treatment group based on crown presence. This is relevant since clinical situations exist where practitioners lack time or expertise to effectively place interdental wire fixation. In clinical situations where CO treatment is the sole form of fixation applied, efforts should be made to preserve the tooth crown of M1 provided it will not compromise healing.

The Stout multiple loop wiring technique provides the benefit of anchoring the wire around multiple teeth on either side of the fracture. Neither clinical nor research investigations have evaluated the impact that the number of teeth incorporated into an interdental wiring technique has on stabilization. Experimental use of bovine rib as a model for mandibular fracture repair demonstrated no significant resistance to breakage existed with greater than three screws in each fracture fragment (24). It is unknown if the number of teeth incorporated in interdental wiring for fracture repair contribute to the inherent strength of the repair device when composite splinting is included.

When confronted with mandibular fracture repair involving M1, careful consideration should be given to which repair techniques can be applied and the impact that removing the tooth may have on that repair. IWC constructs demonstrate significantly greater strength compared to CO or TSC when the crown of M1 is removed. Maintaining M1 significantly increases stiffness in the IWC and TSC constructs and likely is associated with the composite spanning shorter distances between anchor teeth. Fixation devices with greater ultimate strength and stiffness are expected to reduce micromotion. The increased rigidity of the construct resultant from the preservation of the crown of M1 is expected to facilitate a stronger construct that would improve conditions conducive to primary bone healing. The use of transmucosal screws and ligature wire with composite appears to offer no increases in fixation strength or stiffness compared to the other tested fixation techniques. It is unknown how the application of transmucosal screws and ligature wire with composite may enhance stabilization of non-invasive fixation devices in edentulous patients or patients with oligodontia.

## Conflict of Interest Statement

The authors declare that the research was conducted in the absence of any commercial or financial relationships that could be construed as a potential conflict of interest.

## Supplementary Material

The Supplementary Material for this article can be found online at http://journal.frontiersin.org/article/10.3389/fvets.2015.00018

## Footnotes

^1^ScanX Dental Radiography, AllPro Imaging, Melville, NY, USA

^2^Mini driver; Linvatec Hall Surgical, Largo, FL, USA

^3^K100, 15.0 mm Sagittal Blade (0.3 mm, thick); Omega Surgical Instruments, Inc. Gand Blanc MI, USA

^4^700 cross cut fissure bur; Henry Schein Inc, Melville, NY, USA

^5^24 g Orthopedic wire; Miltex, Plainsboro NJ, USA

^6^2.0 mm IMF Screw; DePuy Synthes, Monument, CO, USA

^7^2.0 mm IMF Screw; KLS Martin, Jacksonville, FL, USA

^8^Etch-Rite Dental Etching Gel, 38% phosphoric acid gel 12 mL; Pulpdent, Watertown, MA, USA

^9^Maxi Temp HP 50 mL. Henry Schein Inc, Melville, NY, USA

^10^Bondo; 3M, Maplewood, MN, USA

^11^MTS Bionix 858; MTS Systems Corp, Eden Prairie, MN, USA

^12^*R* Project for Statistical Computing, Vienna, Austria

## Abbreviations

CNTL, control; CO, composite only; IMF, intermaxillary fixation; IWC, Stout interdental wiring and composite; M1, first molar tooth; TSC, transmucosal screw and composite.
