# Supplementary material for: Crown Preservation of the Mandibular First Molar Tooth Impacts the Strength and Stiffness of Three Non-Invasive Jaw Fracture Repair Constructs in Dogs
Source: Front Vet Sci. 2015 Jul 17;2:18. doi: 10.3389/fvets.2015.00018 (PMC4672188; doi:10.3389/fvets.2015.00018)
Supplement: Supplementary file 1 [file Table_1.DOCX]

***Supplementary Material***

**Crown preservation of the mandibular first molar tooth impacts strength and stiffness of three noninvasive jaw fracture repair constructs in dogs**

**Charles Lothamer, Christopher J Snyder*, Sarah Duenwald-Kuehl, John Kloke, Ronald P McCabe, Ray Vanderby Jr.**

*** Correspondence:** Corresponding Author: csnyderdvm@gmail.com

## Supplementary Table 1

**Supplementary Table 1. Fixation weight, time for interdental wire or transmucosal screw and wire placement, time for composite application for each treatment group.**

Mean weights (g) of fixation, time (sec) for wire and composite application are reported. Mean weight of fixation and time for composite application did not vary between treatments or groups, time required for interdental wire application was greater than transmucosal screw and wire application.

| Method of Fixation | Mean Weight of Fixation (range) | Mean Time for Interdental Wire or Transmucosal Screw Application (range) | Mean Time for Composite Application (range) |
| --- | --- | --- | --- |
| CO – Crown Removed | 2.6 g (1.7-4.0) | - | 77 sec (65-101) |
| CO- Crown Present | 2.4 g (1.7-2.9) | - | 80 sec (70-96) |
| TSC - Crown Removed | 2.8 g (1.3-3.9) | 179 sec (149-203) | 82 sec (54-96) |
| TSC- Crown Present | 2.9 g (2.5-3.8) | 176 sec (143-219) | 80 sec (67-100) |
| IWC- Crown Removed | 2.9 g (2.4-4.0) | 456 sec (335-555) | 78 sec (55-114) |
| IWC- Crown Present | 2.7g (1.7-4.4) | 485 sec (390-585) | 77 sec (64-104) |
