# Supplementary material for: Crown Preservation of the Mandibular First Molar Tooth Impacts the Strength and Stiffness of Three Non-Invasive Jaw Fracture Repair Constructs in Dogs
Source: Front Vet Sci. 2015 Jul 17;2:18. doi: 10.3389/fvets.2015.00018 (PMC4672188; doi:10.3389/fvets.2015.00018)
Supplement: Supplementary file 2 [file Table_2.DOCX]

***Supplementary Material***

**Crown preservation of the mandibular first molar tooth impacts strength and stiffness of three noninvasive jaw fracture repair constructs in dogs**

**Charles Lothamer, Christopher J Snyder*, Sarah Duenwald-Kuehl, John Kloke, Ronald P McCabe, Ray Vanderby Jr.**

*** Correspondence:** Corresponding Author: csnyderdvm@gmail.com

## Supplementary Table 2

**Supplementary Table 2. Mean specimen age (months), body weight (kg) and mandible weight (g) for each treatment group.** Within each treatment group mean ages (months), body weights (kg) and specimen weights (g) did not vary between treatment groups. (CO= composite only, TSC=transmucosal screw and wire with composite, IWC=interdental wire and composite, CNTL= control)

| Method of Fixation | Mean Age (range) | Mean Total Body Weight (range) | Weight of mandible prior to fixation (range) |
| --- | --- | --- | --- |
| CO | 18.5 m (11-25) | 9.8 kg (8.4-13.3) | 25.6 g (18.3-36.9) |
| TSC | 20.6 m (13-29) | 9.5 kg (7.0-11.6) | 21.9 g (18.4-33.4) |
| IWC | 24 m (19-30) | 10.3 kg (7.6-11.2) | 26.1 g (18.0-35.4) |
| CNTL | 19.3 m (8-31) | 10.0 kg (7.7-11.6) | 29.4 g (20.7-40.5) |
